# Loss of Ezrin triggers mitochondrial dysfunction and oxidative stress, associated with neuronal cell death

**DOI:** 10.1038/s41420-025-02790-5

**Published:** 2025-10-27

**Authors:** Giuliana Giamundo, Iolanda Carratù, Chiara Barone, Rossella De Cegli, Giovanna Trinchese, Maria Pina Mollica, Dario Antonini, Ivan Conte

**Affiliations:** 1 Department of Biology, University of Naples Federico II, Strada Vicinale Cupa Cintia, 26, Naples, 80126 Italy; 2 Telethon Institute of Genetics and Medicine, Via Campi Flegrei 34, Pozzuoli, Naples 80078 Italy

**Keywords:** Mechanisms of disease, Cell death

## Abstract

EZRIN is a key player in assembling and coordinating molecular signaling, acting as a linker between receptors in plasma membrane and the actin cytoskeleton. High EZRIN expression level has been extensively studied and often associated with metastasis and cancer progression. Recent reports independently suggested associations between Ezrin and mitochondrial alterations or apoptotic processes, the mechanism by which Ezrin modulates these events remain largely unclear. Here we report that the lack of EZRIN-mediated EGFR internalization and translocation on mitochondria is critical for mitochondrial metabolism. Ezrin-deficient (Ezrin^−/−^) cells exhibit marked impairments in mitochondrial respiratory chain (MRC) activity. These cells also show significantly reduced ATP production and elevated mitochondrial ROS levels, revealing cell metabolism deficit. Furthermore, Ezrin loss induces mitochondrial ROS-mediated apoptosis. In vivo, Medaka fish lacking Ezrin display neuronal cell death associated with inflammation, which appear linked to the compromised mitochondrial metabolism and oxidative stress. Our findings reveal a key mechanism within endo-lysosomal signaling that involves Ezrin and the EGFR/TSC complex. both of which are essential for neuronal homeostasis. In conclusion, our data identify a novel molecular pathway in which the Ezrin/EGFR axis regulates mitochondrial metabolism, thereby supporting cellular energy balance and promoting neuronal cell survival.

## Introduction

The epidermal growth factor receptor (EGFR) is expressed in a wide variety of tissues and plays a pivotal role in the proliferation and differentiation of epithelial cells. Binding of epidermal growth factor (EGF) to EGF receptor triggers the receptor dimerization, activates the tyrosine kinase, brings on the phosphorylation of the tyrosine residues of the receptor, causing the activation of signaling events [1]. Besides the activation of different pathways, the activation of EGFR by EGF changes the localization of receptor, promoting the internalization of EGFR, through clathrin -dependent and -independent endocytosis [2]. The internalized receptor accumulates in early endosomes from where they are either recycled back to the cell surface or sent to the late endosome and lysosome for degradation [3]. EGFR has also been found in Golgi, endoplasmic reticulum, nuclei and mitochondria. Importantly, EGFR translocates to mitochondria through interaction with cytochrome *c* oxidase (Cox) subunit II [4], where was subjected to regulation by autophagy [5]. EGFR expression at high levels can activate the apoptotic process [6], preventing autophagic cell death by maintaining the intracellular glucose level [7]. The EGFR-mediated activation of apoptosis requires STAT1 activation followed by induction of the cyclin-dependent kinase inhibitor p21^WAF1/CIP1^ [8]. Importantly, EGFR activation may trigger apoptotic pathways that either promote caspase-1 expression or proceed via caspase-independent mechanisms [9]. EGFR activation can stimulate cellular proliferation in cells expressing low to moderate levels of the receptor, whereas its overexpression can induce apoptotic signaling [10]. Indeed, homodimerization and/or heterodimerization with ErbB-2, strongly potentiates apoptotic process in a variety of cells [11]. Elevate levels of EGFR and ErbB-2 results in activation of Bax and its docking to the mitochondrial membrane. Bax translocation leads to the loss of mitochondrial membrane potential and apoptotic cell death [10]. Importantly, Gamou and Shimizu were the first to demonstrate that treatment of human squamous carcinoma cells with hydrogen peroxide enhances EGFR phosphorylation, thereby establishing a link between oxidative stress and EGFR activation [12]. In addition, the EGFR kinase domain contains six Cys residues, with Cys797 located in the ATP-binding pocket and targeted by endogenous hydrogen peroxide [13]. Recently, we demonstrated a previously unidentified role for Ezrin, a cytoskeleton scaffold protein, in the activation of endosomal signaling network involving EGFR pathways, which regulates TSC complex localization and activation. Internalization of EGFR upon EGF stimuli occurs mainly due to Ezrin binding, that facilitates dimerization and activation of EGFR, resulting in their recruitment to endosomes, followed by AKT activation, that targets and inhibits the TSC complex, modulating the autophagic process [14]. Ezrin, a cytoskeletal linker protein, is an essential member of the ERM (Ezrin-Radixin-Moesin) family, playing a fundamental role in the connection between the plasma membrane and the actin cytoskeleton [15]. This connection is pivotal for maintaining cell shape, polarity, and the dynamic reorganization of the cell surface, which are all critical for various cellular processes, including signal transduction, migration, and adhesion [16]. Ezrin contributes to cell survival by modulating signal transduction pathways that inhibit apoptotic processes. It interacts with a variety of membrane receptors and intracellular signaling molecules, thereby influencing cell survival mechanisms. For instance, Ezrin has been implicated in the activation of the PI3K/Akt pathway, a key axis in promoting cell viability and resistance to apoptosis [17]. Moreover, Ezrin is also able to regulate cell cycle, through the control of cyclins and cyclin-dependent kinases (CDKs) [18]. This function plays a significant role in cell proliferation, regulating the activity of growth factor receptors and adhesion molecules. In this way, Ezrin facilitates the transmission of signals from the extracellular matrix to intracellular space, promoting cell growth. This has a strong impact in various types of cancer [19]. In addition to its well-characterized functions at the plasma membrane, emerging evidence suggests that Ezrin is also involved in mitochondrial regulation, thereby extending its impact on cellular homeostasis. Ezrin modulates mitochondrial morphology by interacting with mitochondrial membranes, which may influence the organelle’s architecture and subcellular localization [20]. Moreover, Ezrin regulates also cell survival and stress response, by regulating apoptotic pathways. Through its association with mitochondrial membranes, indeed, Ezrin can influence the release of apoptotic factors and the integrity of mitochondrial membranes [21]. Elucidating how Ezrin affects mitochondrial morphology, distribution, and function can provide valuable insights into the cellular mechanisms that govern energy metabolism, stress response, and cell survival. Here, we identify a new role for Ezrin in controlling mitochondrial metabolism, examining how these interactions influence mitochondrial shape and distribution. We also investigate the impact of Ezrin on mitochondrial bioenergetics by assessing changes in ATP production, mitochondrial membrane potential, and reactive oxygen species (ROS) generation in cells. Moreover, we analyze how Ezrin affects the balance between mitochondrial fission and fusion, which is essential for mitochondrial quality control and adaptation to metabolic demands. In conclusion, this paper provides evidence indicating EZRIN as a key factor for the communication between mitochondrial bioenergetics pathway and apoptosis, in which EZRIN-mediated EGFR mitochondrial translocation is needed for mitochondrial metabolism and cell survival. Thus, elucidating the regulatory mechanisms governing the EZRIN/EGFR axis and its link with mitochondrial metabolism could suggest novel therapeutic strategies, particularly in the context of cancer. Moreover, given emerging evidence linking this axis to broader cellular stress responses and survival pathways, its modulation may also hold promise in addressing select aspects of neuronal cell death where such mechanisms are perturbed.

## Results

### Ezrin regulates mitochondrial morphology

**Fig. 1:**
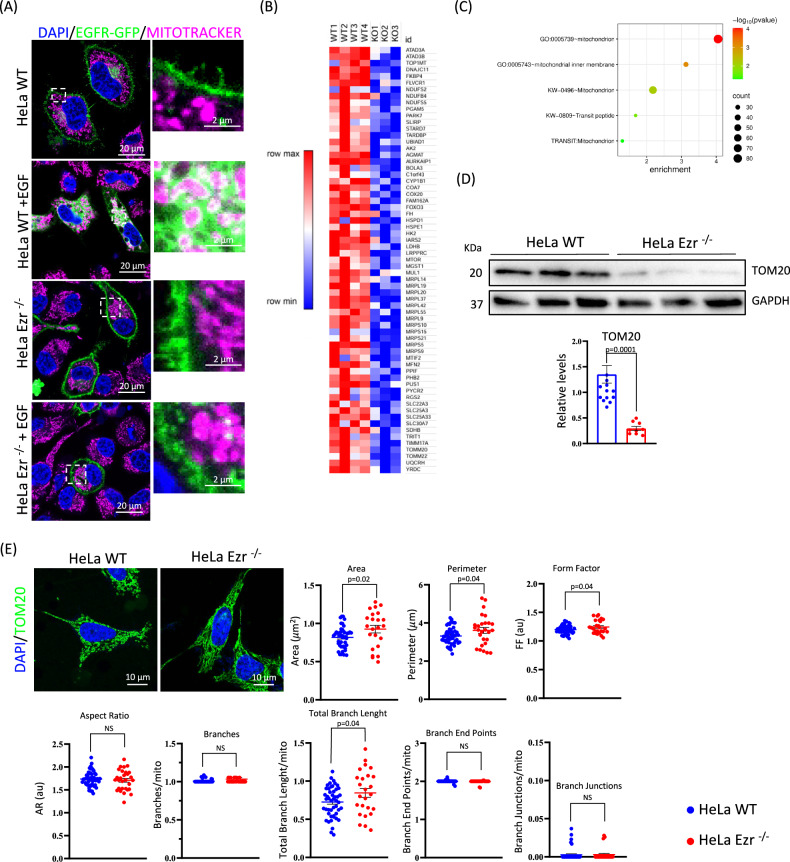
Ezrin deletion affects mitochondrial morphology. **A** Confocal microscopy images showing HeLa WT and EZR^−/−^ cells incubated with the fluorescent dye MitoTracker (magenta) and DAPI (blue) after EGFR-GFP transfection (green). Following EGF stimulation, EGFR translocates to the cytosol and colocalizes with mitochondria in HeLa WT, whereas in HeLa EZR^−/−^ cells remains on the plasma membrane even after EGF stimulation. **B** Heatmap representing the 82 downregulated genes in HeLa EZR^−/−^ cells (FDR ≤ 0.05) involved in mitochondrial processes. **C** Bubble plot representing the biological process associated to downregulated genes in HeLa EZR^−/−^ cells. **D** HeLa WT and EZR^−/−^ cells were lysed and immunoblotted with anti-TOM20 antibody. GAPDH was used as a loading control. The graph shows TOM20 levels relative to GAPDH ± SEM (at least *n* = 3 experiments). Statistical test: unpaired t-test. **E** HeLa WT and EZR^−/−^ cells were cultured in 6-well plates for 24 h. They were then fixed and immunostained with the TOM20 antibody (green) and DAPI (blue). The graphs were generated using the Mitochondria Analyzer plugin in ImageJ, allowing for a detailed analysis of mitochondrial structure ± SEM (at least *n* = 3 experiments). Statistical test: unpaired t-test.

**Fig. 2:**
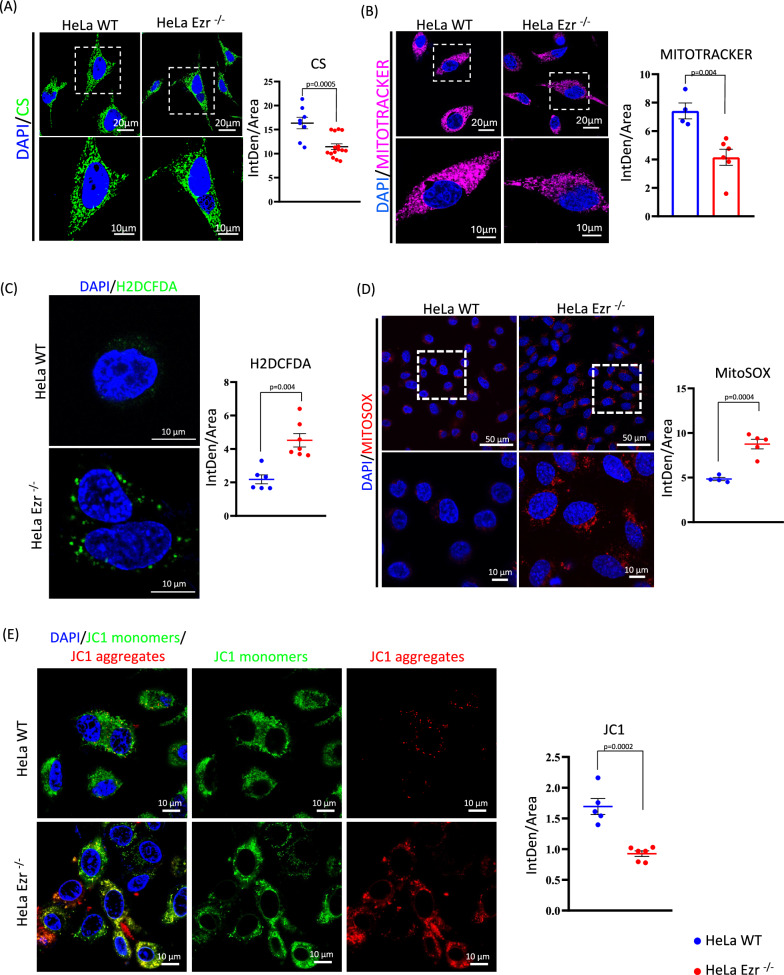
Ezrin regulates ROS clearance and mitochondrial membrane potential. **A** Immunofluorescence analysis of WT and EZR^−/−^ HeLa cells stained with the CS antibody (green) and DAPI (blue) reveals a reduction in CS signal in HeLa EZR^−/−^ cells. The graph shows fluorescence values, measured as Fluorescence Intensity/Area, in WT and EZR^−/−^ cells ± SEM (at least *n* = 3 experiments). Statistical test: unpaired t-test. **B** Confocal microscopy images showing HeLa WT and EZR^−/−^ cells incubated with the fluorescent dye MitoTracker (magenta) and DAPI (blue) with a reduction of mitochondria activity in HeLa EZR^−/−^ cells. The graph shows the fluorescence intensity of MitoTracker-positive cells ±SEM (at least *n* = 3 experiments). Statistical test: unpaired t-test. **C** Immunofluorescence for H2DCFDA. WT and EZR^−/−^ HeLa cells were cultured in 6-well plates for 24 h. They were then incubated with the fluorescent dye H2DCFDA (green) and DAPI (blue). The graph on the right shows the fluorescence intensity of H2DCFDA-positive cells ±SEM (at least *n* = 3 experiments). Statistical test: unpaired t-test. **D** WT and EZR^−/−^ HeLa cells were incubated with the fluorescent dye MitoSOX (red) and DAPI (blue). The graph on the right shows the fluorescence intensity (IntDen/Area) of MitoSOX-positive cells ±SEM (at least *n* = 3 experiments). Statistical test: unpaired t-test. **E** Representative images of immunofluorescence with JC1 dye (green/red) and DAPI (blue) in WT and EZR^−/−^ HeLa cells. The graph on the right shows data expressed as the red/green fluorescence intensity ratio ±SEM (at least *n* = 3 experiments). Statistical test: unpaired t-test.

Recently, we demonstrated that Ezrin facilitates dimerization and activation of EGFR, resulting in its internalization and activation from cytoplasmic membrane to endosomes, followed by AKT activation that targets and inhibits the TSC complex [14]. Considering this, we decided to evaluate if loss of Ezrin further alters the translocation of EGFR on mitochondria upon EGF stimulation. Notably, loss of Ezrin markedly impaired EGFR mitochondrial localization upon EGF treatment in HeLa Ezrin^−/−^ (Ezr^−/−^) cells compared to wild-type controls (Fig. 1A). Given the pivotal role of EGFR on mitochondrial membrane for mitochondria dynamics and function, we sought to investigate the biological relevance of this phenotype by analyzing a possible alteration of mitochondria pathway. Therefore, we used comparative analysis by unbiased Quant-seq on HeLa Ezr^−/−^ cells [14]. The transcriptomics analysis identified a large group of genes, downregulated in the absence of Ezrin, enriched in mitochondrial pathways (Fig. 1B, C, and Supplementary Table S1). Consistent with this, western blot analysis revealed that HeLa Ezr^−/−^ cells displayed a reduction of outer mitochondrial membrane TOM20 protein (Fig. 1D). Furthermore, as TOM20 protein is a widely used marker of mitochondrial morphology [22], immunofluorescence analysis revealed that HeLa Ezr^−/−^ cells displayed an increased elongation of mitochondria, as assessed by quantification of TOM20 fluorescence (Fig. 1E). Notably, quantitative analysis of mitochondrial area, perimeter and form factor revealed an increase of these parameters in HeLa EZR^−/−^ cells compared to controls (Fig. 1E), suggesting an alteration of mitochondria structures consistent with enhanced mitochondrial elongation. Concordantly, Total Branch Length graph, representing the ramifications length, supported this hypothesis. Total Branch analysis of mitochondria further indicated major elongation and ramification in absence of EZRIN (Fig. 1E). Notably, an increase in elongation of mitochondria may indicate a defect in mitochondrial function, resulting from an imbalance in fusion and fission dynamics. In physiological conditions, mitochondria continually undergo dynamic fusion and fission events to preserve cellular health. Disruption of this balance, characterized by impaired fission or excessive fusion, can lead to elongated and extensively branched mitochondrial networks. Such morphological changes may reflect cellular stress responses, attempts to compensate for organelle damage, or failures in mitophagy, the selective autophagic clearance of dysfunctional mitochondria. These abnormalities are frequently documented in pathologies such as neurodegenerative diseases, cancer, and metabolic disorders, where mitochondrial dysfunction plays a significant role in pathogenesis [23]. Thus, to establish whether the increased mitochondrial elongation was dependent on mitochondrial dysfunction, we decide to investigate by immunofluorescence the distribution of citrate synthase (CS) enzyme, which is crucial to produce ATP, through the synthesis of NADH and FADH in the mitochondria. Notably, the data show a significant reduction of fluorescence intensity of CS in HeLa EZR^−/−^ compared to control cells (Fig. 2A), supporting a mitochondrial dysfunction. Consistently, we observed a decrease in MitoTracker staining, an indicator of mitochondrial dysfunction (Fig. 2B) associated with mitochondrial depolarization, which can result from oxidative stress, impaired electron transport chain function, or abnormalities in mitochondrial dynamics.

### Ezrin controls ROS production and mitochondrial membrane potential

To gain further insight into the deleterious events caused by the loss of Ezrin action on mitochondrial function, we monitored mitochondrial ROS production in HeLa EZR^−/−^ compared to control cells. Interestingly, we observed an increase of ROS production in HeLa EZR^−/−^ cells by live-imaging studies with fluorescence of H2DCFDA (2’, 7’ -dichlorodihydrofluorescein diacetate), a cell-permeable probe used to detect intracellular ROS (Fig. 2C). To gain insight into the ROS production, we took advantage of the MitoSOX superoxide indicator, which specifically targets mitochondrial superoxide. Concordantly to the hypothesis of a mitochondrial dysfunction, high levels of mitochondrial ROS fluorescence were observed in HeLa EZR^−/−^ compared to control cells (Fig. 2D). Altogether the data above indicate a new role for Ezrin in maintaining cellular redox balance via mitochondrial homeostasis. ROS accumulation in mitochondria can induce a rapid depolarization of inner mitochondrial membrane potential and subsequent impairment of oxidative phosphorylation [24]. We asked if the increased ROS levels in mitochondria could cause an alteration of membrane potential. To test this hypothesis, the mitochondrial membrane potential was investigated by JC-1 live-imaging study, that exhibits a shift in emission from green to red as the mitochondria membrane potential becomes more polarized. Notably, we observed a strong reduction of mitochondrial membrane potential in HeLa EZR^−/−^ compared to control cells (Fig. 2E). In summary, these findings

show that Loss of EZRIN causes mitochondrial dysfunction by affecting mitochondrial structure and function.

### Loss of Ezrin alters mitochondrial ATP production

Mitochondrial membrane depolarization and ROS accumulation have a negative effect on mitochondrial oxidative phosphorylation system (OXPHOS), causing the block of ATP synthesis due to the lack of energy needed to couple the protons flow with ATP production [25]. To gain further insight into the mitochondrial dysfunction, we monitored the activities of mitochondrial complexes.

**Fig. 3:**
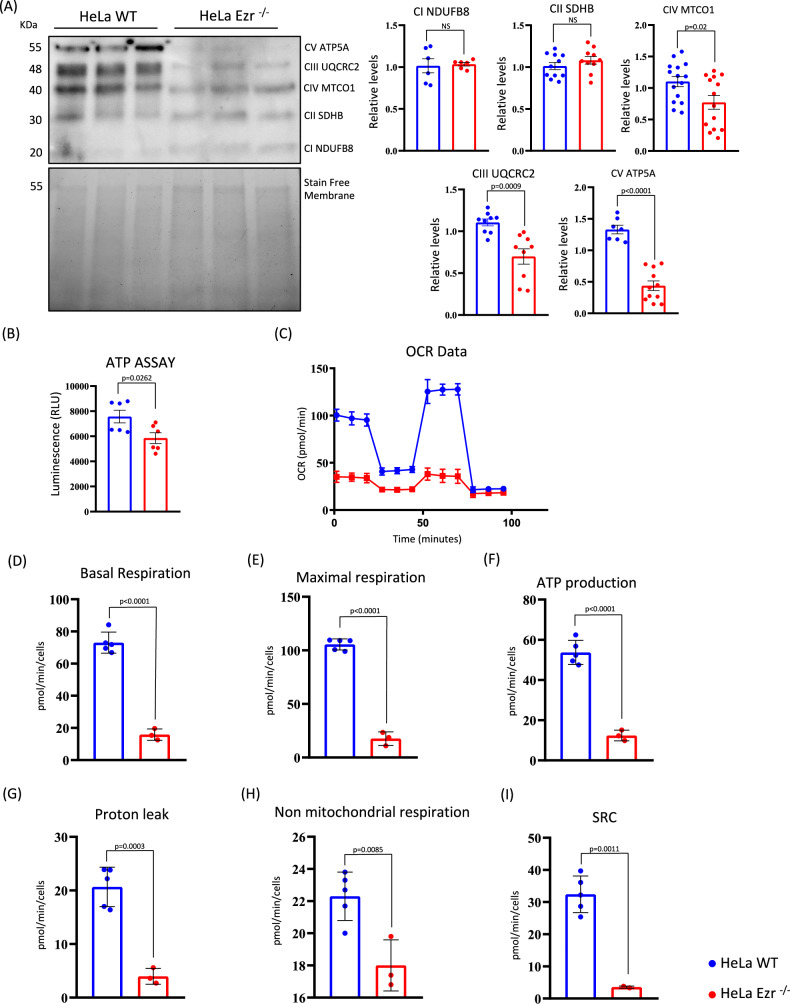
Ezrin deletion causes mitochondrial respiratory chain disruption and ATP depletion. **A** WT and EZR^−/−^ HeLa cells were lysed and the proteins incubated with an antibody against OXPHOS. Bottom, the membrane activated prior to antibody incubation is shown as a loading control. The graphs show the quantification of the different OXPHOS complexes relative to the membrane ±SEM (at least *n* = 3 experiments). Statistical test: unpaired t-test. **B** The graph shows the relative ATP luminescence levels ± SEM (at least *n* = 3 experiments) in HeLa WT and HeLa EZR^−/−^. Statistical test: unpaired t-test. **C**–**I** Representative graph of Cell Mito Stress assay performed by Seahorse XFp analyzer in WT and EZR^−/−^ HeLa cells is reported (**C**). In the bar charts, each point in the OCR time courses is the average of three technical replicates. Basal respiration (**D**), maximal respiration (**E**), ATP production (**F**), proton leak (**G**) non-mitochondrial respiration (**H**) and spare respiratory capacity (SRC) (**I**) are reported. The values are expressed as means ± SD.

As showed in Fig. 3A, western blot analysis revealed a significant reduction of “ATP producers”, namely complexes III, IV and V, in HeLa EZR^−/−^ compared to control cells. In contrast, complexes I and II, which primarily mediate NADH production and exhibit lower sensitivity to ROS accumulation, remained unaltered. (Fig. 3A). To determine whether the reduction of complexes III, IV and V induced an impairment of ATP production, we quantified intracellular ATP levels. Notably, a significant reduction of ATP synthesis was observed in HeLa EZR^−/−^ cells compared to control cells (Fig. 3B), demonstrating the inefficiency of oxidative metabolism.

Moreover, we tested the oxygen consumption rates in HeLa WT and EZR^−/−^ cells. We observed a significant reduction of all parameters related to mitochondrial metabolism in HeLa EZR^−/−^ compared to control cells. Notably, basal (Fig. 3D) and maximal (Fig. 3E) respiratory rates were significantly reduced in HeLa EZR^−/−^ cells, indicating a reduction in mitochondrial oxidative capacity. Consequently, the ATP production was significantly reduced (Fig. 3F). Accordingly, the proton leakage and the non-mitochondrial respiration were significantly lower in HeLa EZR^−/−^ compared to control cells (Fig. 3G, H). Similarly, spare respiratory capacity was also significantly reduced in HeLa EZR^−/−^ cells (Fig. 3I).

### Mitochondrial dysfunction is associated to increased mitophagy in HeLa EZR^−/−^ cells

**Fig. 4:**
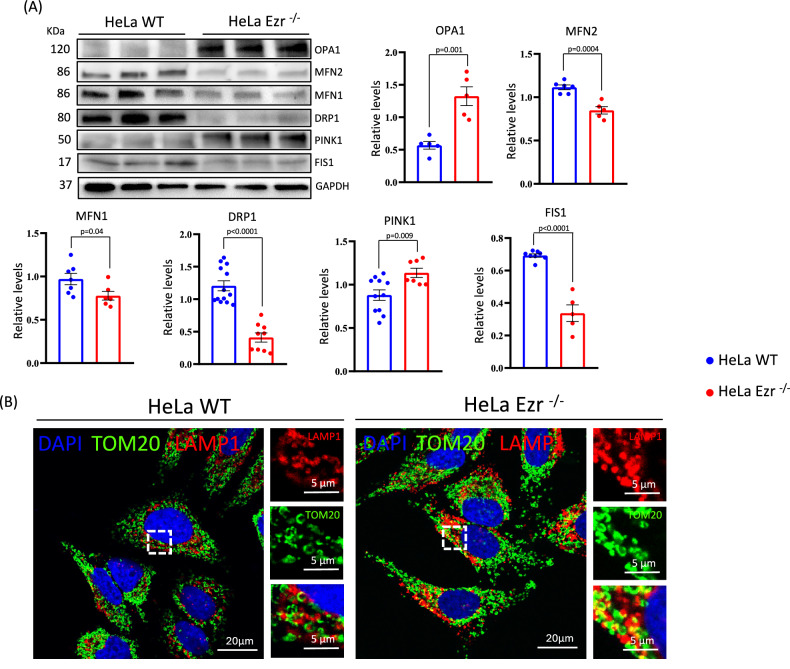
Analysis of fusion, fission, and mitophagy in the absence of Ezrin. **A** WT and EZR^−/−^ HeLa cells were lysed, and proteins were incubated with anti-TOM20, anti-OPA1, anti-MFN2, anti-MFN1, anti-DRP1, anti-PINK1, and anti-FIS1 antibodies. GAPDH was used as a loading control. The graphs show the levels of OPA1, MFN2, MFN1, DRP1, PINK1, pPARKIN, PARKIN, FIS1, and TOM20 relative to GAPDH ± SEM (at least *n* = 3 experiments). Statistical test: unpaired t-test. **B** Immunofluorescence analysis of WT and EZR^−/−^ HeLa cells stained with LAMP1 (red), TOM20 (green), and DAPI (blue) antibodies reveals increased mitophagy, as indicated by the colocalization of TOM20 and LAMP1 signals in the absence of EZRIN.

Since dysfunctional mitochondria initiate mitophagy through several key signals, primarily involving mitochondrial membrane depolarization, oxidative stress, and protein modifications, we evaluated if this metabolic impairment was accompanied by an altered mitochondrial turnover. To this end, we assessed several critical processes involved in mitochondrial quality control, including fusion, fission, intracellular transport, and mitophagy. These mechanisms collectively ensure proper mitochondrial renewal, which is essential to meet the high energy demands of cells and maintain cell homeostasis. Failure to maintain this energy supply due to impaired mitochondrial quality control contributes to the neuronal cell death in neurodegenerative disorders [26]. Interestingly, we observed a significant increase of internal membrane fusion marker OPA1, accompanied by a significant decrease of external membrane fusion markers MFN1 and MFN2 in HeLa EZR^−/−^ cells compared to control (Fig. 4A). An increase in OPA1, which regulates inner mitochondrial membrane fusion, alongside a decrease in MFN1 and MFN2, which mediate outer membrane fusion, suggests a compensatory mechanism where inner membrane fusion is enhanced to maintain mitochondrial integrity despite reduced outer membrane fusion. The latter condition is often observed in mitochondria stress conditions [27]. Notably, we also found a reduction of fission proteins DRP1 and FIS1 (Fig. 4A), suggesting a decrease of fission process between mitochondria. These findings suggested a possible alteration in the ratio between fission versus fusion of mitochondria. Indeed, a reduction in DRP1 and FIS1 can affect mitophagy. These proteins are essential for mitochondrial fission, a mechanism that separates damaged mitochondria for clearance. When their expression decreases, mitochondrial fragmentation is limited, which may hinder the efficient elimination of dysfunctional mitochondria through mitophagy. However, under specific stress conditions, diminished DRP1 and FIS1 activity can result in mitochondrial elongation, a state that has been associated with an increase in mitophagy [28]. To gain insight into the intricate relationship between dysfunctional mitochondria and mitophagy, we evaluated the effect of loss of Ezrin on mitophagy process. We observed an increase of PINK1 protein, in HeLa EZR^−/−^ compared to control cells (Fig. 4A), suggesting a possible increase of mitophagy. In accordance with this hypothesis, by using TOM20 and LAMP1 immunofluorescence assay we found that EZR^−/−^ cells had a significant increased number of lysosomes containing mitochondria, indicative of augmented mitophagy process (Fig. 4B). Taken together, these findings show that loss of Ezrin alters mitochondria function and increases mitophagy to remove damage mitochondria.

### Loss of Ezrin promotes programmed cell death in HeLa cells

**Fig. 5:**
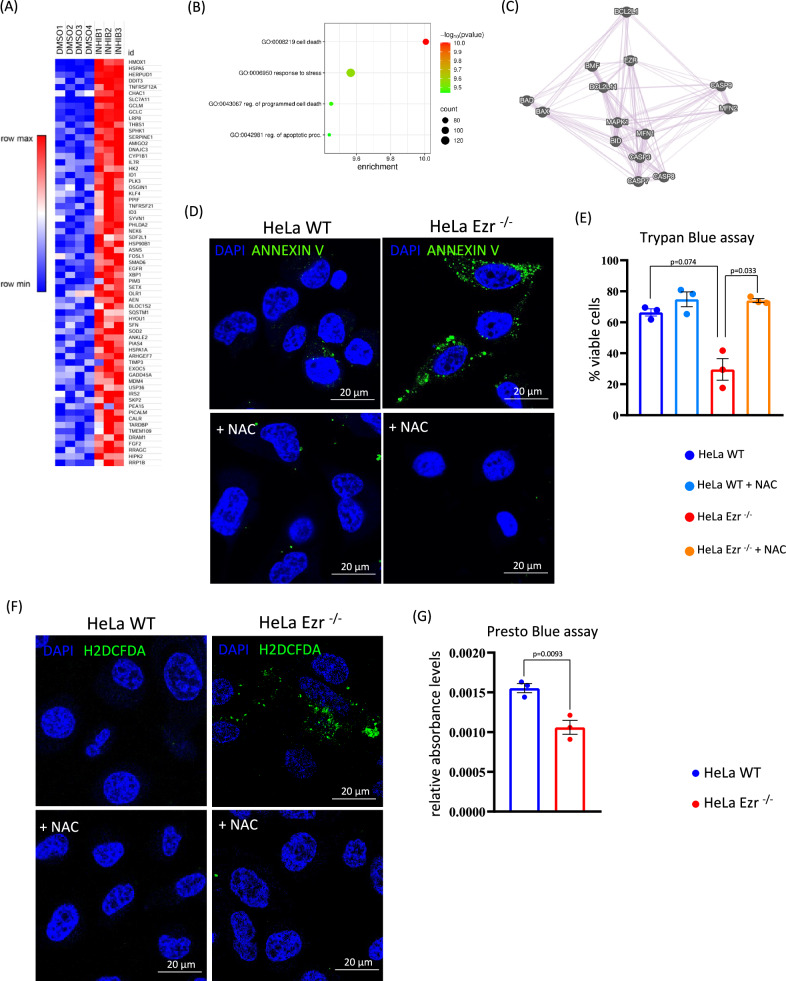
NAC treatment reduces ROS accumulation and cell death in HeLa EZR^−/−^ cells. **A** Heatmap representing the 65 upregulated genes in HeLa treated with *NSC668394* (FDR ≤ 0.05) involved in cell death and apoptotic processes. **B** Bubble plot representing the biological process associated to upregulated genes in HeLa treated with *NSC668394*. **C** Co-expression, obtained by GeneMANIA, highlight Ezrin and apoptotic protein binding. **D** HeLa WT and HeLa EZR^−/−^ were cultured in 6-well plates for 24 h. Subsequently, they were treated with an Annexin V/Propidium Iodide mix for 20 min and DAPI (blu). The images show an increase in cell death in HeLa EZR^−/−^ compared to HeLa WT, while NAC drastically reduces cell death in HeLa EZR^−/−^. **E** HeLa WT and EZR^−/−^ cells were cultured in 6-well plates for 24 h. They were then treated with 1.5 mM NAC for 1 h and subsequently trypsinized. A mix of Trypan Blue and cells was prepared and counted using the LUNA cell counter. The graph shows the individual viability percentages for HeLa WT, HeLa WT + NAC, HeLa EZR^−/−^, and HeLa EZR^−/−^ + NAC ± SEM (at least *n* = 3 experiments). Statistical test: unpaired t-test. **F** HeLa WT and EZR^−/−^ cells were cultured in 6-well plates for 24 h. They were then treated with 1.5 mM NAC for 1 h and incubated with the fluorescent dye H2DCFDA (green) and DAPI (blue). The images show ROS reduction when NAC is used in HeLa EZR^−/−^. **G** HeLa WT and EZR^−/−^ cells were cultured in 96-well plates for 24 h. They were then treated with Presto blue reagent. The graph shows a reduction in relative absorbance levels percentage in HeLa EZR^−/−^ cells compared to HeLa WT, reflecting a lower cell viability in HeLa EZR^−/−^ cells.

To assess the biological relevance of our findings, we investigated whether enhanced mitochondrial dysfunction may contribute to an induction of apoptosis in absence of Ezrin [29]. Thus, we carried out quantitative-transcriptome analysis on HeLa cells upon *NSC668394* treatment, a specific Ezrin inhibitor [30]. Differential expression analysis revealed a statistically significant group of upregulated genes in HeLa treated with *NSC668394*, enriched in cell death and apoptotic processes (Fig. 5A, B, and Supplementary Table S2). Interestingly, gene network based on co-expression interaction further revealed EZRIN as a possible direct partner for both mitochondrial and related apoptotic proteins (Fig. 5C). Thus, we postulated that loss of EZRIN induced mitochondrial stress signaling and this in turn activates a transcriptional program that promotes apoptosis in HeLa cells. We assessed the biological relevance of this pathway by quantifying Annexin V-positive cells. Notably, a significant increase in Annexin V-positive cells was observed in the absence of EZRIN compared to controls, consistent with enhanced apoptosis. Most importantly, this increase was completely rescued by treatment with N-Acetyl-L-Cysteine (NAC), a small-molecule antioxidant that mitigates oxidative stress [31], thereby establishing a functional link between mitochondrial dysfunction, ROS accumulation, and apoptotic cell death (Fig. 5D). Concordantly, as showed in Fig. 5E, Trypan Blue assays revealed a marked reduction in cell viability (~30%) upon EZRIN depletion, which was significantly restored (~70%) following NAC treatment (Fig. 5E), supporting the link between ROS accumulation and cell death in HeLa EZR^−/−^ cells. Moreover, live-imaging assay with H2DCFDA showed a rescue in NAC-treated HeLa EZR^−/−^ compared to untreated HeLa EZR^−/−^ cells (Fig. 5F). Since mitochondrial dysfunction can significantly impact cell proliferation, we further observed a significant reduction of cell proliferation in HeLa EZR^−/−^ cells compared to control cells (Fig. 5G). Collectively, these results indicate that EZRIN loss contributes to apoptosis, at least in part, via mitochondrial dysfunction and the resulting increase in oxidative stress.

### Lack of Ezrin induces mitochondrial dysfunction and cell death in medaka fish

**Fig. 6:**
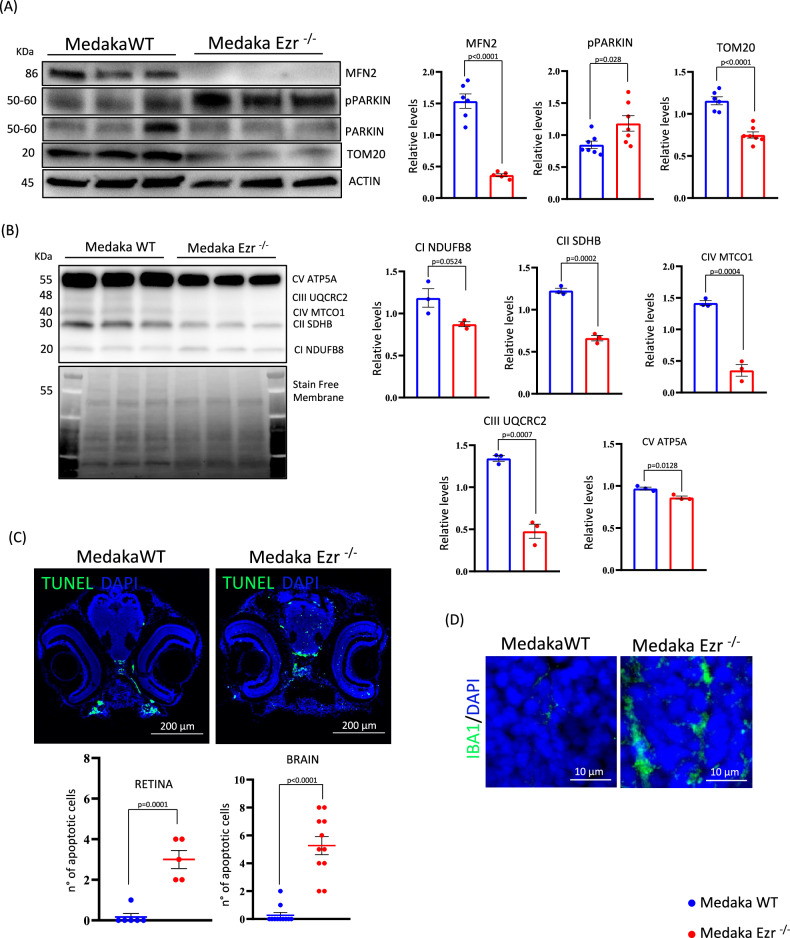
Ezrin deletion causes mitochondrial dysfunction, increased cell death and inflammation. **A** Proteins extracted from stage 40 larvae of Medaka WT and Ezr^−/−^ were incubated with antibodies against MFN2, pPARKIN, PARKIN, and TOM20. ACTIN was used as a loading control. The graphs show the levels of MFN2, pPARKIN, PARKIN, and TOM20 normalized to ACTIN ± SEM (at least *n* = 3 experiments). Statistical test: unpaired t-test. **B** Medaka WT and Ezr^−/−^ larvae were lysed and proteins were incubated with the OXPHOS antibody. On the left, the membrane activated prior to antibody incubation is shown as a loading control. The graphs show the quantification of the different OXPHOS complexes relative to the membrane ±SEM (at least *n* = 3 experiments). Statistical test: unpaired t-test. **C** Immunofluorescence images of Medaka WT and Ezr^−/−^ for TUNEL (green) and DAPI (blue). The graphs below show the number of apoptotic cells in the brain and retina of Medaka, respectively. **D** Stage 40 larvae of Medaka WT and Ezr^−/−^ sections were immunostained with anti-IBA1 (green) antibody and DAPI (blue). Confocal microscopy images show increased inflammation in Medaka Ezr^−/−^compared to Medaka WT.

To further get insight on the link between Ezrin and mitochondrial dysfunction, we carried out in vivo experiments. Thus, we used a recently homozygous Ezrin mutant Medaka fish (*Oryzias latipes*, Ol), in which Ezrin protein was depleted [14]. Notably, the observed phenotype in this model closely resembles that of EZRIN knockout mice [32], providing a relevant vertebrate system to investigate EZRIN’s functional role in mitochondrial biology. To determine whether the *olEzrin*^−/−^ medaka phenotype was indeed related to abnormal mitochondrial function, we analyzed mitochondrial structure and function. Notably, western blot analysis confirmed the reduction of TOM20, indicating the block of cytosolic protein import in the mitochondrial matrix (Fig. 6A). Moreover, the fusion protein Mfn2 also showed the same trend observed in EZR^−/−^ HeLa cells, demonstrating the reduction of mitochondrial fusion process in *olEzrin*^−/−^ medaka (Fig. 6A). Additionally, as observed in HeLa cells, we discovered a failure of electron transport chain through the western blot performed on OXPHOS in Medaka fish (Fig. 6B). The data showed a strong reduction of complexes II, III, IV and V in *olEzrin*^−/−^ medaka compared to control, suggesting an impairment of respiratory chain to sustain a powerful energetic metabolism. Consistent with these observations, we observed an increased pParkin level in *olEzrin*^−/−^ medaka, supporting the enhancement of mitophagy to remove damaged mitochondria (Fig. 6A). These results are in line with the evidence emerged in cells, confirming that Ezrin can regulate mitochondrial metabolism and mitophagy. Considering the key role of Ezrin in controlling ROS production at mitochondrial level, we decided to verify whether ROS production affected cellular survival in *olEzrin*^−/−^ medaka models. We reasoned that if Ezrin directly controls cell survival via mitochondrial metabolism, loss of Ezrin should induce similar in vitro phenotype because of alterations in apoptosis. Interestingly, *olEzrin* deletion caused a significant increase of apoptotic cells in both brain and retina of *olEzrin*^−/−^ Medaka compared to wild-type (Fig. 6C), tissues where EZRIN is abundantly expressed [33]. Thus, we hypothesized that Ezrin-mediated EGFR mitochondrial trafficking could participate in the homeostasis of neuronal cells. Since mitochondrial dysfunction can precede microglial activation and contribute to neuroinflammation, particularly in conditions involving neuronal cell death, we decided to test the effect of Ezrin-mediated mitochondrial dysfunction on microglia, by using immunofluorescence staining with a specific antibody against Ionized calcium binding adaptor molecule 1 (Iba1). As shown in Fig. 6D, the thalamic area of *olEzrin*^−/−^ Medaka brain showed an increased Iba1 signal, confirming the link with Ezrin and inflammation process. Collectively, these observations strongly suggest that the regulation of mitochondrial homeostasis by Ezrin is conserved across the evolution and might play a key role in neuronal cell survival. Additional research is necessary to elucidate the clinical relevance of our findings, with the aim of developing targeted therapeutic strategies that inhibit cancer progression driven by Ezrin overexpression and restore Ezrin function in pathological conditions characterized by dysfunction of mitochondria metabolism in neuronal cell death.

## Discussion

EGFR is traditionally known for its role in cell signaling at the plasma membrane. However, recent studies have revealed that EGFR can also translocate from plasma membrane into the mitochondria, influencing cellular metabolism, apoptotis and drug resistance in cancer cells [34]. Notably, EGFR can be localized on both the outer and inner mitochondrial membranes. This localization appears independent from the endocytosis process and linked to cell survival, as its mitochondrial EGFR presence is reduced when cells undergo apoptosis [35]. Moreover, EGFR translocation on mitochondria has been observed in response to stress signals, suggesting a potential mechanism for drug resistance in cancer therapy [36]. Interestingly, mitochondrial EGFR has been implicated in regulating mitochondrial dynamics, particularly in non-small-cell lung cancer (NSCLC). It promotes mitochondrial fission, enhances ATP production, and increases cell motility, thereby contributing to cancer metastasis. Furthermore, EGFR’s interaction with mitochondrial proteins such as Mfn1 disrupts mitochondrial fusion, further supporting its role in cancer progression [37]. However, the molecular mechanisms that guarantee EGFR translocation to mitochondria remain largely unknown. Recently, we demonstrated that cytoskeleton protein Ezrin is essential for EGFR endocytosis and trafficking on endosomes. Endosomal EGFR activation occurs mainly through EZRIN interaction that induces dimerization and activation of EGF-EGFR complex, resulting in their recruitment on endosomes, followed by EGFR endosomal signaling [14]. Here, we provide evidence that Ezrin plays a new central role in mitochondrial metabolism. We show that depletion of Ezrin is critical for mitochondrial function, as its deletion impinged on mitochondrial respiratory chain, resulting in a compromised of ATP production associated with ROS generation. These results confirm that Ezrin works as a scaffold protein for EGFR, favoring its activity on different organelles. We show that depletion of Ezrin was mirrored by lack of EGFR translocation on mitochondria, affecting their structure. The altered mitochondria structure could depend on different mechanisms, including altered fusion and fission resulting in a major elongation of these organelles. Indeed, mitochondrial shape depends by balancing the opposing processes of fusion and fission; unbalanced fission leads to mitochondrial fragmentation, and unbalanced fusion leads to mitochondrial elongation. These processes allow mitochondria to interact with each other, ensuring their function [38]. Considering these, our data sustain that loss of EZRIN might cause an increase of mitochondria fusion, reducing the fragmentation process of fission. As the fine-tune balance of mitochondrial dynamicity is associated with cancer and neurodegenerative diseases, our findings highlight the potential relevance of Ezrin in tuning the mitochondrial activity in cell homeostasis and survival. In accordance with this scenario, we show that depletion of Ezrin is critical for excess of mitochondrial ROS production as previously suggested [39]. Notably, the increase of ROS as negative feedback strongly affects the mitochondrial membrane, causing an increase of membrane permeability, and in turn enhancing the proton re-entry, deteriorating the oxidative phosphorylation. Our findings further reveal that loss of Ezrin causes a reduction of respiratory chain, resulting in a strong depletion of ATP production and compromising the cellular energy request. Interestingly, an increase in mtROS production can induce mitophagy, a selective degradation of mitochondria, to suppress the production of ROS and to minimize the number of damaged mitochondria [40]. Because we recently showed that Ezing controls autophagy pathway by modulating EGFR signaling [14], these findings suggest that the Ezrin-mediated regulation of EGFR signaling could simultaneously be required for modulating the mitochondrial function and their clearance via mitophagy pathway. Notably, we provide evidence that loss of Ezrin induces enhancement of mitophagy process; while mitophagy is essential for removing damaged mitochondria, its uncontrolled activation can disrupt mitochondrial homeostasis and promoting apoptosis [41] including in neuronal cells [42]. Our in vitro data on Ezrin-mediated uncontrolled mitochondria dysregulation and mitophagy represents a driving force for tuning apoptosis pathways. These data shed light on the impact of mtROS production induced by depletion of EGFR signaling and its responsiveness to NAC treatment, supporting the key role of ROS accumulation in cell survival. Importantly, the potential applications of our newly identified molecular network extend beyond the comprehensive and highly relevant identification of mitochondrial homeostasis regulation pathways in neuronal cells. Recently, several attempts have been made to develop therapeutic pharmacological approaches to treat cancers in which Ezrin is overexpressed and sustain tumors growth and metastasis. Our study shows the efficacy of a chronic depletion of Ezrin function, via genetic and pharmacological repression of Ezrin, for blocking EGFR signaling and mitochondrial homeostasis in vitro and in vivo, which could represent an important therapeutic strategy to treat cancers associated with EGFR signaling and/or Ezrin overexpression the most common cause of breast cancer [43, 44]. Beyond this, our findings also revealed that lack of Ezrin alters microglial reactivity associated with an increase of neuronal cell death. These findings suggest that Ezrin might participate as a molecular bridge linking mitochondrial signaling to neuronal cells. However, the precise mechanisms by which mitochondrial metabolism interfaces with EGFR signaling in distinct physiological and pathological neuronal contexts remain to be fully elucidated, needing additional studies.

## Material and methods

### Ethics approval and consent to participate

The Ezrin^−/−^ medaka (*O. latipes*) line was previously described [14]. The cab strain of wild-type and Ezrin^−/−^ medaka lines were maintained following standard conditions (i.e., 12 h/12 h dark/light conditions at 27 °C) and embryos were staged was previously described [14]. All animal procedures were reviewed and approved by the Ethics Committee of the TIGEM Institute (Pozzuoli, NA, Italy). Experiments involving fish were conducted in full accordance with institutional animal welfare guidelines and were authorized by the Italian Ministry of Health (authorization no. 7B56B.0), Department of Public Health, Animal Health, Nutrition and Food Safety, in compliance with Legislative Decree 26/2014 governing animal experimentation.

### Dissecting the transcriptomic effect of EZR deletion and of the EZR inhibition

Two unbiased RNA-seq analysis have been performed, the first was on *EZR-*deleted-HeLa stable clones compared to WT cells and the second on the same cells treated with *NSC668394* compared to DMSO treatment. The threshold for the statistical significance of gene expression was FDR < 0.05.

1328 differentially expressed genes (DEGs) were found in EZR_KO samples compared to WT cells (GSE288769), while 672 DEGs were found following the inhibition (GSE288768).

Gene Ontology enrichment analysis (GOEA) was performed on inhibited and induced genes, separately by using the DAVID Bioinformatic tool [45, 46] restricting the output to Biological Process (BP), Cellular Compartments (CC) terms (refer to Supplementary Table). The ‘Kyoto Encyclopedia of Genes and Genomes’ (KEGG Pathway) analyses was also performed [47, 48]. The threshold for statistical significance of GOEA was FDR < 0.1 and Enrichment Score ≥1.5, while for the KEGG Pathway analyses was FDR < 0.1.

### Library preparation

Total RNA was quantified using the Qubit 2.0 fluorimetric Assay (Thermo Fisher Scientific). Libraries were prepared from 125 ng of total RNA using the NEGEDIA Digital mRNAseq research grade sequencing service (NEGEDIA srl) which included library preparation, quality assessmentand sequencing on a NovaSeq 6000 sequencing system using a single-end, 100 cycle strategy (Illumina Inc.). Illumina NovaSeq 6000 base call (BCL) files were converted in fastq file through bcl2fastq. Alignment was performed with STAR 2.6.0a. The expression levels of genes were determined with HTseq-counts 0.9.1. The genome build is Hg38. The raw data were analyzed by NEGEDIA srl proprietary NEGEDIA Digital mRNAseq pipeline (v2.0) which involves a cleaning step by quality filtering and trimming, alignment to the reference genome and counting by gene (sourceforge.net/projects/bbmap/) [49, 50]. The raw expression data were normalized, analyzed and visualized by Rosalind HyperScale architecture (https://cran.r-project.org/web/packages/fpc/index.html) [51] (OnRamp BioInformatics, Inc.).

### Functional annotation analysis

Functional annotation analysis of differential regulated genes (DEGs) (FDR ≤ 0.05) was performed using ShinyGO (https://bioinformatics.sdstate.edu/go/), and DAVID (https://davidbioinformatics.nih.gov/). Heatmap was generated using Morpheus (https://software.broadinstitute.org/morpheus), and Bubble plots using SRplot (https://www.bioinformatics.com.cn/plot_basic_enrichment_bubble_plot_126_en).

### Accession code

The transcriptomics data have been deposited in NCBIs Gene Expression Omnibus (GEO) [52] and are accessible through GEO Series accession number. GEO Series accession number and title of the datasets are the following: GSE288768 - “Transcriptomic analysis of Hela Ezrin inhibited cells”, and GSE288769 - “Transcriptomic analysis of Hela Ezrin Ko cells”.

### Cell cultures and treatments

HeLa WT cell lines were obtained from the American Type Culture Collection (ATCC), while HeLa EZR^−/−^ cells were previously generated in our laboratory [14]. HeLa cells were cultured in Dulbecco’s Modified Eagle Medium (DMEM) (Euroclone) supplemented with 10% (v/v) fetal bovine serum (FBS) and 5% penicillin-streptomycin. All cell lines were cultured in a 37 °C humid incubator with 5% CO_2_ following the suppliers’ guidelines.

For Bafilomycin treatment, 250.000 HeLa WT and HeLa EZR^−/−^ cells were seeded in 6-well plates. After 24 h, 200 nM Bafilomycin diluted in DMEM was added for 3 h. Cells were then fixed in 4% paraformaldehyde (PFA) in 1X PBS (Chem Cruz SC-281692).

For N-acetylcysteine (NAC) treatment, 250.000 HeLa WT and HeLa EZR^−/−^ cells were seeded on coverslips in 6-well plates. After 24 h, 1.5 mM NAC diluted in DMEM was added for 1 h.

For EGFR-GFP transfection 250.000 HeLa WT and HeLa EZR^−/−^ cells were seeded in a 6-well plate on coverslips. After 24 h, cells were transfected with 2.5 µg of EGFR-GFP vector (Addgene, 32751), using Opti-MEM (GIBCO, Reduced Serum Medium, 2482110) and Lipofectamine 2000 (Invitrogen Thermo Fisher Scientific, 2343141) mix, following the manufacturer’s protocol. After 24 h, EGF stimulation was obtained with 10 ng/ml of animal-free recombinant human EGF (Peprotech AF-100-15) for 3 h.

### Trypan blue assay

200.000 HeLa WT and HeLa EZR^−/−^ cells were seeded in duplicate in 6-well plates (4 wells total). After 48 h, one well of HeLa WT and one well of HeLa EZR^−/−^ were treated with NAC as previously described; the other two wells served as controls. All cells (HeLa WT, HeLa EZR^−/−^, HeLa WT + NAC, HeLa EZR^−/−^ + NAC) were trypsinized and resuspended in DMEM. A mix of 10 μl resuspended cells and 10 μl Trypan Blue 0.4% (Logos Biosystems T13001) was prepared for each condition and counted using the LUNA-II™ Automated Cell Counter (Logos Biosystems L40001).

### PrestoBlue assay

30.000 HeLa WT and HeLa EZR^−/−^ cells were seeded in a 96-well plate containing 100 µL/well of cell culture medium. Cells were incubated for 24 h in a 37 °C incubator using the appropriate CO_2_ percentage. Subsequently, the volume of cell viability reagent was added 1/10^th^ directly to cells in culture medium according to the protocol (PrestoBlue™ HS Cell Viability Reagent, ThermoFisher Scientific A13261). Then, cells were incubated for 10 min at 37 °C in a cell culture incubator, protected from direct light and the absorbance was read at 570 nm using a Synergy HTX Multi-Mode Reader (BioTek, DE39680027).

### ATP assay

One million HeLa WT and HeLa EZR^−/−^ cells were washed twice in PBS (Dulbecco’s Phosphate-Buffered Saline, Euroclone), trypsinized, and incubated for 5 min. Cells were then centrifuged at 1000 rpm for 5 min and resuspended in 1× Passive Lysis Buffer. The assay was performed according to the manufacturer’s protocol for the *ATP Determination Kit* (Invitrogen Thermo Fisher Scientific, A22066). A reaction mix was prepared containing 20× Buffer, 0.1 M DTT, 10 mM D-luciferin, and Luciferase stock solution. The cell suspension was combined with the reaction mix and loaded into 96-well plates for 15 min. ATP production was measured as luminescence using a Synergy HTX Multi-Mode Reader (BioTek, DE39680027).

### Seahorse analysis

Mitochondrial metabolism in HELA cells were assessed by the Seahorse XFp analyzer (Seahorse Biosciences, North Billerica, MA, USA), by using the Cell Mito Stress Test kit (Agilent, Santa Clara, Ca, USA, cat# 103010-100). HELA cells were seeded in Seahorse plates in complete DMEM medium for 12 h (2.0 ×10^4^ cells/well). Before cell mito-stress analyses, the medium was replaced with a buffered base medium (Agilent Seahorse-103575) supplemented with 2 mM glutamine, 1 mM pyruvate and 10 mM glucose at pH 7.4. The plates were equilibrated at 37 °C in a CO_2_ free incubator for 1 h. Basal oxygen consumption rate (OCR) was determined in the presence of glutamine (2 mM) and pyruvate (1 mM). The injections specific for Cell Mito Stress assay were oligomycin (1.5 μM), FCCP (2 μM), and antimycin A/rotenone (0.5 μM). Basal respiration was calculated as difference between OCR before oligomycin injection and non-mitochondrial respiration, determined after the injections of the inhibitors antimycin A/rotenone. The ATP production in the basal state was obtained from the decrease in respiration by inhibition of the ATP synthase with oligomycin. Afterward, the mitochondrial electron transport chain was stimulated maximally by the addition of the uncoupler FCCP (1 µM), and the maximal respiration was calculated as difference between maximum OCR after FCCP injection and non-mitochondrial respiration. The FCCP-stimulated OCR can then be used to calculate spare respiratory capacity, defined as the difference between maximal respiration and basal respiration. Spare respiratory capacity is a measure of the ability of the cell to respond to increased energy demand or under stress. The mitochondrial respiration was expressed as the oxygen consumption rate per minute normalized to the number of cells. In our experimental conditions, the same cell number/well was plated before the OCR measurements; the cell count was obtained by using the Luna cell counter (TwinHelix).

### Annexin-V/propidium iodide assay

A total of 250.000 HeLa WT and HeLa EZR^−/−^ cells were seeded in 12-well plates on coverslips. After 24 h, cells were treated with a mix prepared following the protocol for the Annexin V-FITC/PI Apoptosis Detection Kit (Elabscience, E-CK-A211) and incubated for 20 min. Hoechst (0.001 mg/ml, Thermo Scientific, XL3773331) was added for 10 min. After a brief wash with PBS 1X, cells were visualized under the Olympus FLUOVIEW FV3000 confocal microscope.

### Live assays

HeLa WT and HeLa EZR^−/−^ cells were seeded 24 h prior to treatment on coverslips in 6-well plates. Cells were washed in DMEM without Phenol Red (Gibco) and treated with the following probes:

- H2DCFDA 5 μM (Invitrogen, 2600176)
- MitoSOX Red (Invitrogen, 1942306)
- MitoTracker Deep Red FM 300 nM (Invitrogen, 2528089)
- JC1 5 μg/ml (Invitrogen, 2788100)

Probes were incubated for 30 min, and Hoechst (0.001 mg/ml) was added for 10 min. Cells were washed three times in PBS and imaged with the Olympus FLUOVIEW FV3000 confocal microscope.

### Immunofluorescence

HeLa WT and HeLa EZR^−/−^ cells were seeded on coverslips in 6-well plates. After 24 h, cells were fixed in 4% paraformaldehyde for 15 min at room temperature followed by washing with 1% PBS. To prevent nonspecific antibody binding, cells were blocked with 2.5% BSA, 0.2% Triton in PBS for TOM20 and CS staining, and Blocking Buffer 0.5% (1X PBS, 5% normal serum, 0.3% Triton X-100) for LAMP1 staining. Medaka fish at stage 40 were subjected to anesthesia and then fixed by incubation in 4% PFA for 4 h at room temperature (RT). Samples were rinsed three times with PTW 1X (1X PBS, 0.1% Tween, pH 7.3) and then incubated overnight in 15% sucrose/PTW 1X at 4 °C, and then again incubated overnight in 30% sucrose/PTW 1X at 4 °C and embedded. Sixteen-micrometer cryosection were collected on slides. Cells and sections were incubated overnight at 4 °C with the following primary antibodies: mouse anti-TOM20 (1:50, Santa Cruz, sc-17764), rabbit anti-CS (1:1000, Invitrogen, PA5-22126), rabbit anti-LAMP1 (1:400, Abcam, ab24170), rabbit anti-IBA1 (1:50, Fujifilm, 019-19741). After washing with 1% PBS, cells and sections were incubated with secondary antibodies Alexa 488 goat anti-rabbit/mouse (1:1000, Invitrogen A-11008 rabbit, A-11032 mouse) and DAPI (1:500, Vector Laboratories H-1200) for 1 h at room temperature. Coverslips were then washed in PBS and mounted with PBS/glycerol, and images were acquired with the Olympus FLUOVIEW FV3000 confocal microscope.

### Western blot analysis

Cells were harvested to extract total proteins. Cell samples were lysed using RIPA buffer (150 mM sodium chloride, 1% Triton X-100, 0.5% sodium deoxycholate, 0.1% sodium dodecyl sulfate, 50 mM Tris, pH 8.0) with protease inhibitor cocktail (Thermo Fisher Scientific, 78420). Medaka proteins were collected at developmental stage 40. Larval proteins were extracted using 10 mM Tris HCl buffer + 0.2% SDS with protease inhibitors (Thermo Fisher Scientific, 78420). Protein concentration was determined using a Nanodrop (ND-1000 v3.6.0). Proteins were separated by SDS-PAGE and transferred to PVDF membranes (EMD Millipore, IPVH00010), then blocked with 5% BSA (Tocris 5217) in TBS with 0.1% Tween (TBS-T) for at least 1 h at room temperature. Membranes were incubated overnight at 4 °C with primary antibodies. The following antibodies were used: mouse anti-TOM20 (1:500, Santa Cruz, sc-17764), rabbit anti-FIS1 (1:500, Santa Cruz, sc-98900), rabbit anti-DRP1 (1:500, Santa Cruz, sc-32898), goat anti-OPA1 (1:500, Santa Cruz, sc-30573), rabbit anti-MFN1 (1:500, Santa Cruz, sc-50330), mouse anti-MFN2 (1:500, Santa Cruz, sc-100560), mouse anti-GAPDH (1:1000, Santa Cruz, SC-32233), mouse anti-OXPHOS (1:500, Abcam, ab110413), rabbit anti-actin (1:1000, Cell Signaling #4967), rabbit anti-phospho PARKIN (1:1000, Cell Signaling #O60260), mouse anti-PARKIN (Cell Signaling, 4211), abbit anti-PINK1 (1:1000, Cell Signaling #Q9BXM7), rabbit anti-Citrate synthase (1:1000, Invitrogen PA5-22126). After three washes in TBS-T, membranes were incubated for 1 h at room temperature with HRP-conjugated goat anti-rabbit and anti-mouse secondary antibodies (1:10,000, EMD Millipore, 12-348; 12-349). Western blot detection was performed using the ChemiDoc XRS+ system (Bio-Rad), and quantification was carried out using ImageJ software.

### Animal model and housing conditions

Medaka fish (*Oryzias latipes*) were maintained under standard conditions with a 14 h light/10 h dark cycle at a temperature of 28 °C and pH 7.5. Eggs were collected daily, separated by gently rolling them on paper, and then transferred onto 2% agarose plates in 1× Yamamoto solution (composed of sodium chloride [NaCl], potassium chloride [KCl], calcium chloride [CaCl₂], and sodium bicarbonate [NaHCO₃]). Grooves were previously created in the agarose using a plastic mold to ensure proper accommodation of individual eggs. The eggs were maintained in 1× Yamamoto solution inside an incubator set to 27 °C. Embryos were monitored daily under a stereomicroscope to assess development and phenotype. The Yamamoto solution was regularly replaced, and non-viable embryos were removed to prevent contamination [53]. Adult Medaka fish were kept at 28 °C under a 14 h light/10 h dark cycle. Embryos were prepared and maintained according to the protocol described by Iwamatsu [53], using Yamamoto 1X solution at a constant temperature of 28 °C.

### Genotyping of Ezr^–/–^ Medaka line

The olEzrin knockout line was previously generated in our laboratory [14]. Heterozygous olEzrin fish were crossed (HT × HT), and fertilized eggs were collected and incubated until hatching, typically at stage 40 (9–10 days post-fertilization). For genotyping, a small fragment of the caudal fin was excised from each larva to extract genomic DNA. Larvae were anesthetized using 500 μl of tricaine methanesulfonate (MS-222, Sigma-Aldrich) diluted in 10 ml of Yamamoto 1X solution. DNA extraction was performed using 100 μl of lysis buffer (100 mM Tris-HCl, 200 mM NaCl, 0.2% SDS, 5 mM EDTA) and 1.5 μl of Proteinase K (20 mg/ml, Proteinase K Recombinant PCR Grade, 03115836001). The heads of individual larvae were fixed in 4% paraformaldehyde (PFA) in PTW 2X (PBS 1X, 0.2% Tween-20) for subsequent analyses. The tail-containing samples were incubated overnight at 56 °C for digestion, followed by Proteinase K inactivation at 75 °C for 30 min. DNA was precipitated with decreasing concentrations of ethanol and resuspended in 30 μl of nuclease-free water. DNA concentration was determined using a Nanodrop spectrophotometer (ND-1000 V3.6.0). For PCR, 50 ng of genomic DNA was used per reaction and the following primers were used:

**EZR Forward**: GAACTCCTTCTAGCACCC

**EZR Reverse**: CCGCCTCCCTCCTCAATC

Three distinct PCR products can be obtained depending on the genotype. PCR products were analyzed by agarose gel electrophoresis and visualized using a Geldoc XR+ system (Bio-Rad). The wild-type (WT) larvae showed a single band at 756 base pairs, the olEzrin knockout larvae (Ezr^−/−^) displayed a single band at 386 base pairs, while heterozygous larvae (Ezr^+/–^) exhibited two bands, one at 756 base pairs and one at 386 base pairs.

### TUNEL assay in Medaka fish

Stage 40 Medaka larvae were anesthetized and subsequently fixed by incubation in 4% paraformaldehyde (PFA) for 4 h at room temperature (RT). Samples were rinsed three times with 1X PTW (1X PBS, 0.1% Tween, pH 7.3) and incubated overnight at 4 °C in 15% sucrose/1X PTW, followed by a second overnight incubation at 4 °C in 30% sucrose/1X PTW. For block preparation, larvae were oriented perpendicularly to the mold plane and embedded in a solution of 15% sucrose in 1X PBS (ABP399-1, PBS 10X Solution) and gelatin. The embedded samples were cryopreserved in N-pentane (ThermoScientific, Q26H738) and liquid nitrogen, and then stored at −80 °C for long-term preservation. Transverse cryosections (12 µm thick) were obtained at the level of the optic nerve and collected on Superfrost Plus microscope slides (Fisher Scientific). Slides were fixed in 4% PFA for 15 min, washed in 1X PBS for 30 min, and incubated in a permeabilization solution (0.1% Triton X-100, 0.1% sodium citrate in 1X PBS). Slides were then boiled in citrate buffer (1 M citric acid, 1 M sodium citrate, in H₂O), cooled in 1X PBS, and incubated for 30 min in 0.1 M Tris-HCl, pH 7.5, containing 3% BSA and 20% FBS. After rinsing in 1X PBS, slides were incubated with the enzyme mix as described in the *In Situ Cell Death Detection Kit, Fluorescein* (Sigma-Aldrich, 11684795910) and DAPI (1:500, Vector Laboratories, H-1200) for 1 h at 37 °C. Finally, slides were washed in 1% PBS, mounted in PBS/glycerol, and imaged using an Olympus FLUOVIEW FV3000 confocal microscope.

## Supplementary information

Supplemental Table Legends

Original Western Blots

Table S1

Table S2

## Data Availability

The full length uncropped original western blots are shown in the ‘Supplementary Material’.
