# Supplementary material for: Loss of Ezrin triggers mitochondrial dysfunction and oxidative stress, associated with neuronal cell death
Source: Cell Death Discov. 2025 Oct 27;11:490. doi: 10.1038/s41420-025-02790-5 (PMC12559342; doi:10.1038/s41420-025-02790-5)
Supplement: Supplementary file 1 — Supplemental Table Legends [file 41420_2025_2790_MOESM1_ESM.docx]

**Supplementary Material**

**Supplemental Table Legends**

**Table S1**. **List of genes significantly downregulated upon Ezrin depletion, identified through transcriptomic analysis.** These genes show enrichment in mitochondrial-associated pathways.

**Table S1**. **Differential expression analysis identified a statistically significant set of genes upregulated in NSC668394-treated HeLa cells.** Functional enrichment revealed association with cell death and apoptotic pathways.
