# Supplementary material for: Loss of Ezrin triggers mitochondrial dysfunction and oxidative stress, associated with neuronal cell death
Source: Cell Death Discov. 2025 Oct 27;11:490. doi: 10.1038/s41420-025-02790-5 (PMC12559342; doi:10.1038/s41420-025-02790-5)
Supplement: Supplementary file 2 — Original Western Blots [file 41420_2025_2790_MOESM2_ESM.pptx]

## Slide 1
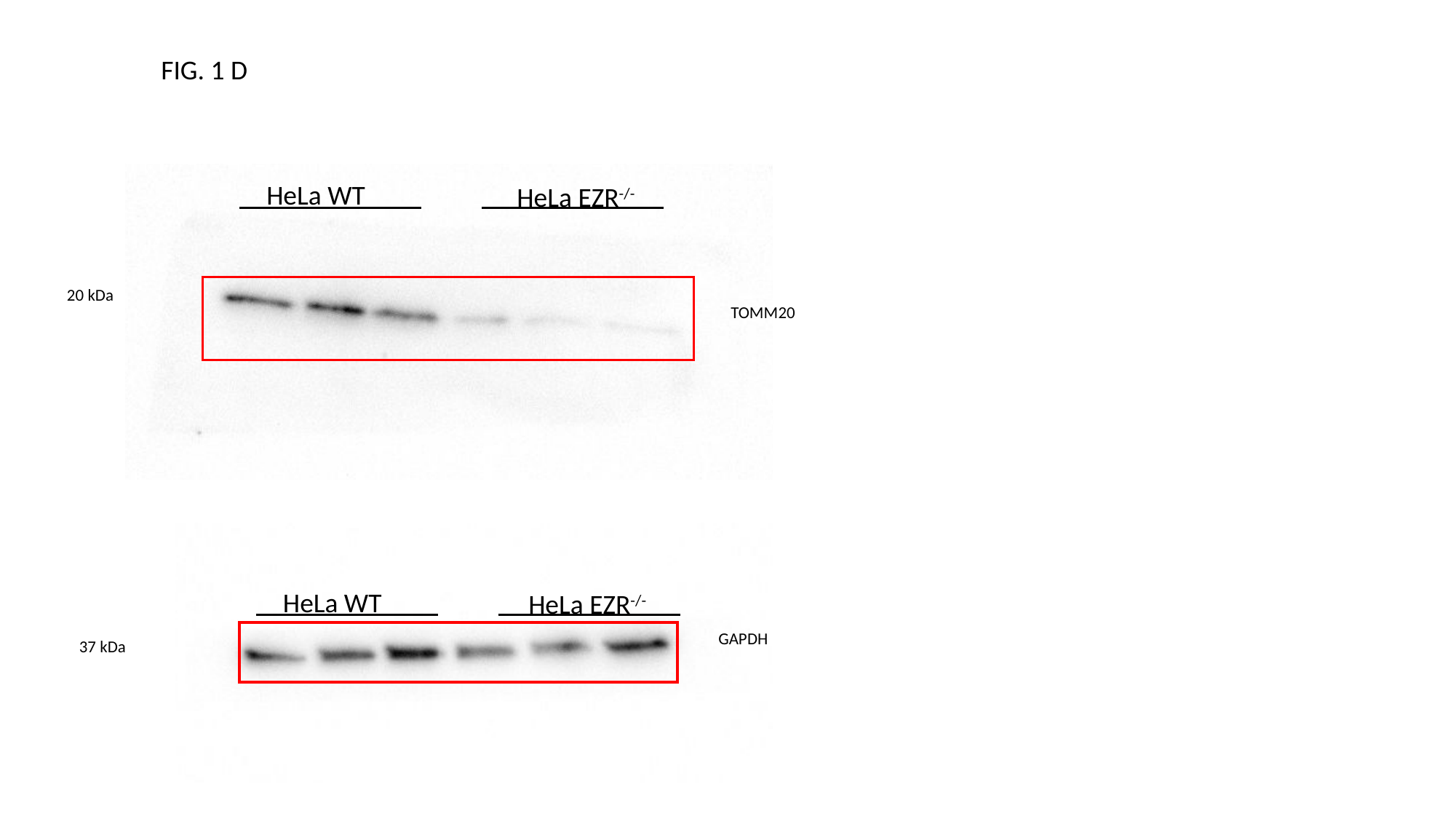

FIG. 1 D
HeLa WT
HeLa EZR-/-
20 kDa
TOMM20
HeLa WT
HeLa EZR-/-
GAPDH
37 kDa

## Slide 2
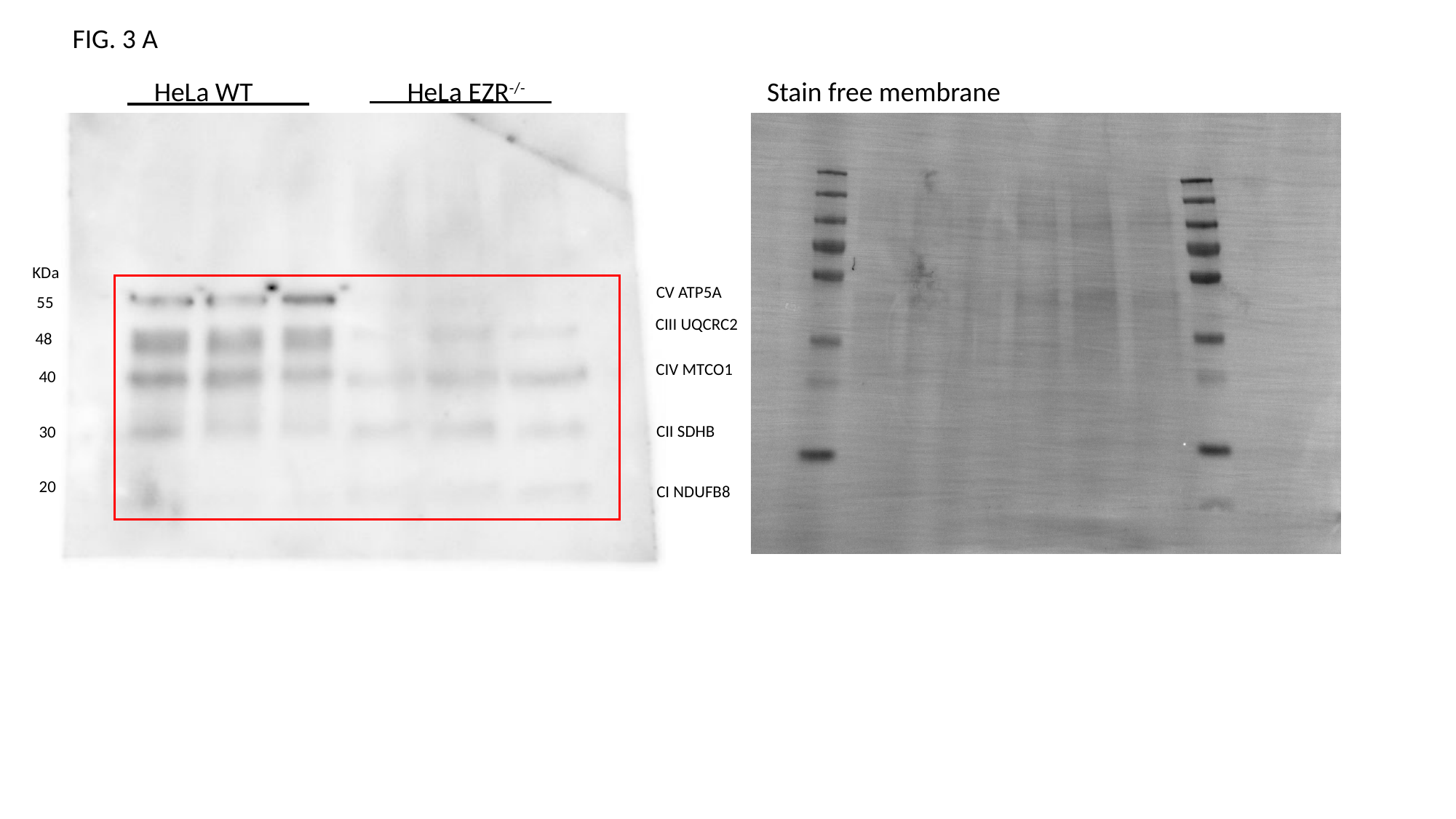

FIG. 3 A
HeLa WT
HeLa EZR-/-
Stain free membrane
KDa
CV ATP5A
55
CIII UQCRC2
48
CIV MTCO1
40
CII SDHB
30
20
CI NDUFB8

## Slide 3
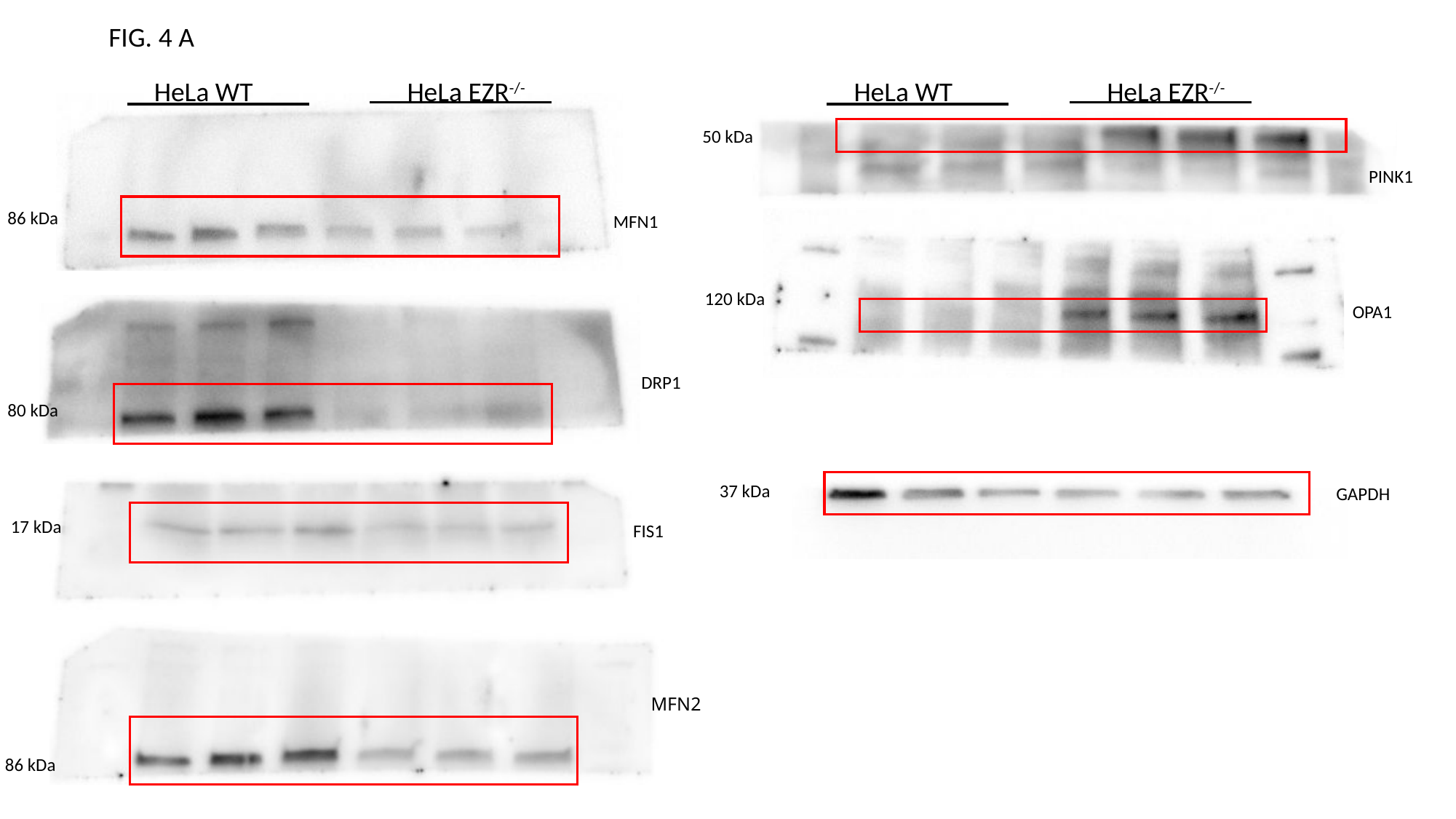

FIG. 4 A
HeLa WT
HeLa EZR-/-
HeLa WT
HeLa EZR-/-
50 kDa
PINK1
86 kDa
MFN1
120 kDa
OPA1
DRP1
80 kDa
37 kDa
GAPDH
17 kDa
FIS1
MFN2
86 kDa

## Slide 4
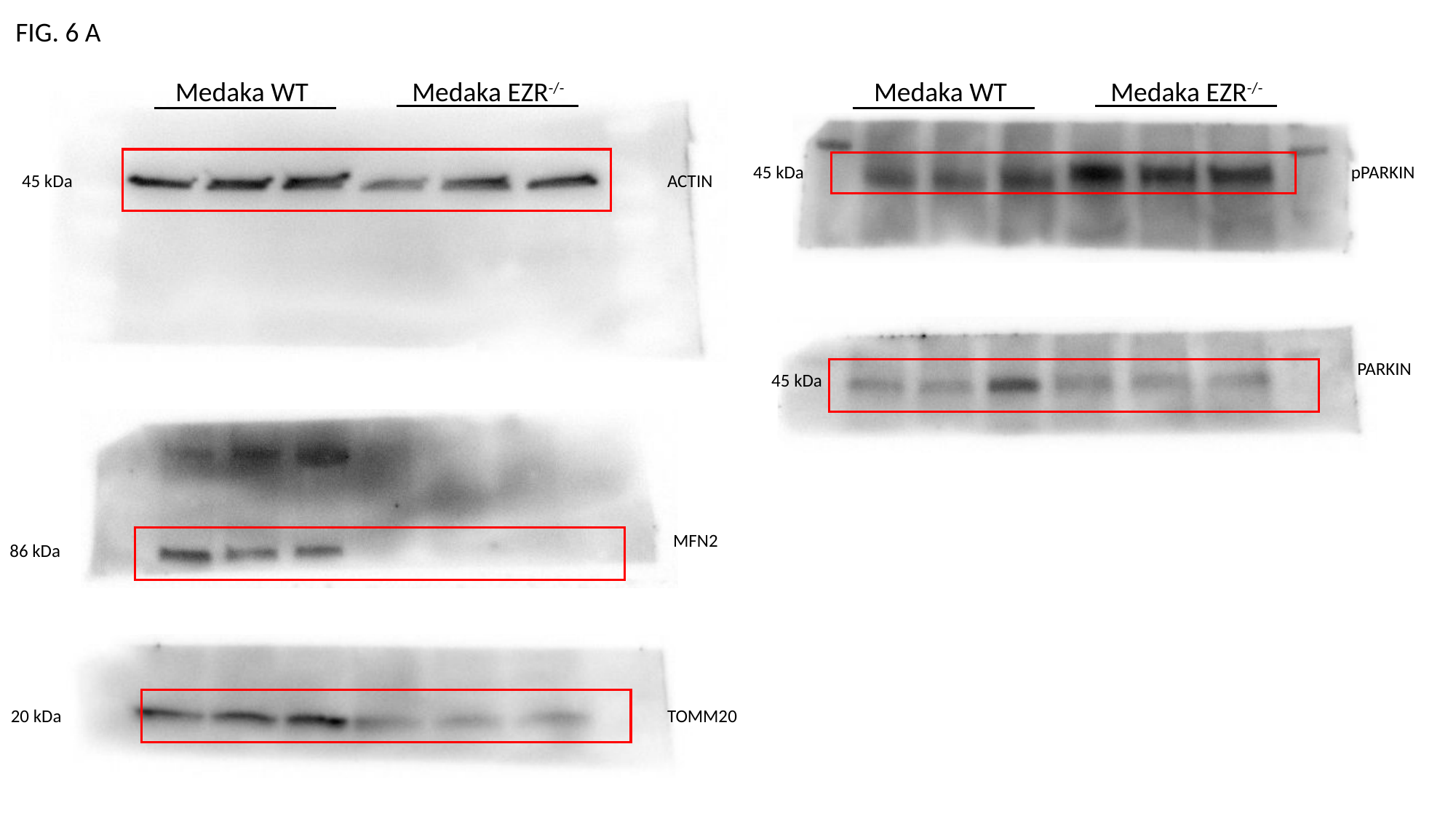

FIG. 6 A
Medaka WT
Medaka EZR-/-
Medaka WT
Medaka EZR-/-
45 kDa
pPARKIN
45 kDa
ACTIN
PARKIN
45 kDa
MFN2
86 kDa
TOMM20
20 kDa

## Slide 5
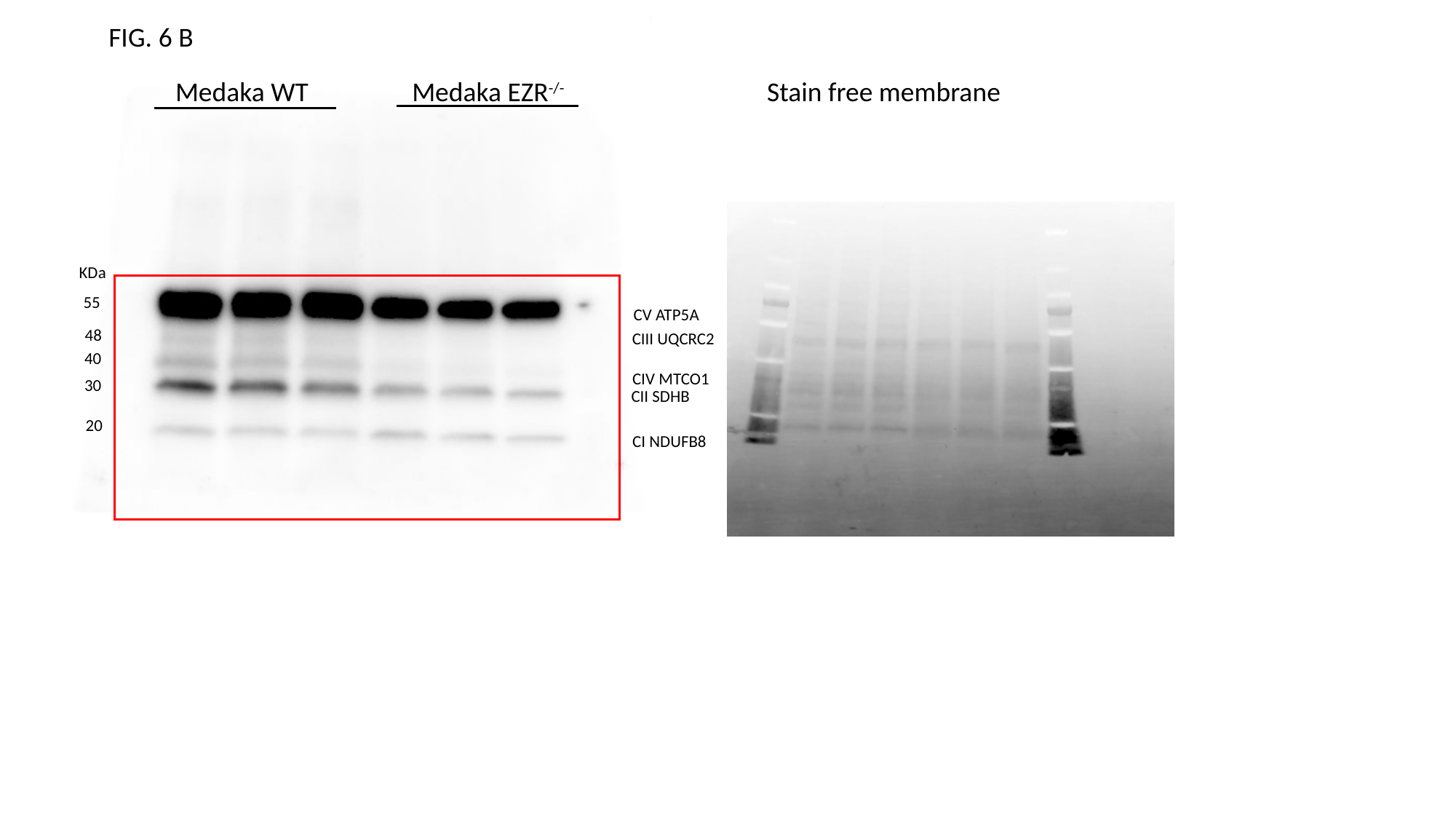

FIG. 6 B
Medaka WT
Medaka EZR-/-
Stain free membrane
KDa
55
CV ATP5A
48
CIII UQCRC2
40
CIV MTCO1
30
CII SDHB
20
CI NDUFB8
